# Nelarabine in T-cell acute lymphoblastic leukemia: intracellular metabolism and molecular mode-of-action

**DOI:** 10.1038/s41375-025-02529-2

**Published:** 2025-02-17

**Authors:** Femke M. Hormann, Sean G. Rudd

**Affiliations:** Science for Life Laboratory (SciLifeLab), Department of Oncology-Pathology, Karolinska Institutet, Stockholm, Sweden

**Keywords:** Chemotherapy, Acute lymphocytic leukaemia

## Abstract

T-cell acute lymphoblastic leukemia (T-ALL) patients often have a poor 5-year event-free survival. The only T-ALL specific drug in clinical practice is nelarabine. A prodrug of the deoxyguanosine analog ara-G, nelarabine is a rationally designed agent selective for the treatment of T-cell malignancies. Originally approved for relapsed/refractory T-ALL, it is increasingly used in T-ALL therapy and is currently being evaluated in upfront treatment. Whilst the clinical use of nelarabine has been the topic of multiple review articles, a thorough overview of the preclinical data detailing the molecular underpinnings of its anti-leukemic activity is lacking, which is critical to inform mechanism-based use. Thus, in the present article we conducted a semi-systematic review of the literature and critically evaluated the preclinical knowledge on the molecular pharmacology of nelarabine. Whilst early studies identified ara-G triphosphate to be the principal active metabolite and nuclear DNA synthesis to be a key target, many fundamental questions remain that could inform upon future use of this therapy. These include the nature of nelarabine-induced DNA lesions and their repair, together with additional cellular targets of ara-G metabolites and their role in efficacy and toxicity. A critical avenue of research in need of development is investigation of nelarabine combination therapies, both in the context of current T-ALL chemotherapy regimens and with emerging anti-leukemic agents, and we highlight some areas to pursue. Altogether, we discuss what we can learn from the preclinical literature as a whole and present our view for future research regarding nelarabine treatment in T-ALL.

## Introduction

T-cell acute lymphoblastic leukemia (T-ALL) is a T-cell malignancy accounting for 15% of (pediatric) acute lymphoblastic leukemia diagnoses [1–3]. Compared with its more common B-cell counterpart, T-ALL patients are generally older, more likely to be male, and present with a higher leukocyte count [4]. T-ALL has a poorer prognosis than B-ALL, with a 5-year event-free survival of ~80% compared to 90% in B-ALL [1–3]. Adult T-ALL has a worse 5-year event-free survival of 65% compared to 82% in pediatric 1-9 year T-ALL and 76% in pediatric 10-17 year T-ALL [5]. T-ALL consists of many different subtypes, with a recent study identifying 15 [6], but whilst in B-ALL subtypes help guide risk stratification, in T-ALL only the early T-precursor ALL is associated with a high-risk and used for risk stratification in some treatment protocols [1, 4]. T-ALL patients have many mutations or alterations for which targeted agents could prove beneficial, such as JAK inhibitors, CDK4/6 inhibitors, or BCL2 inhibitors [4]. However, none of these drugs are currently approved for the treatment of T-ALL. The only T-ALL specific drug that is currently approved is the nucleoside analog nelarabine, which is approved for the treatment of relapsed/refractory T-ALL [7].

An important factor that can inform upon optimal use of any chemotherapeutic agent is a thorough molecular understanding of its metabolism and mode-of-action, in order to inform mechanism-based decisions in the clinic [8]. Nucleoside analogs typically have complex and poly-pharmacologic mechanisms of action [9] and it is becoming increasingly apparent that many gaps remain in our understanding of how these therapies exert their anti-cancer properties. Thus, we conducted a semi-systematic review of the literature regarding nelarabine, and in this article compile the current knowledge on the metabolism and molecular mode-of-action of this therapy, with the aim to highlight critical gaps in our understanding and direct future avenues of research.

## History of development

**Fig. 1:**
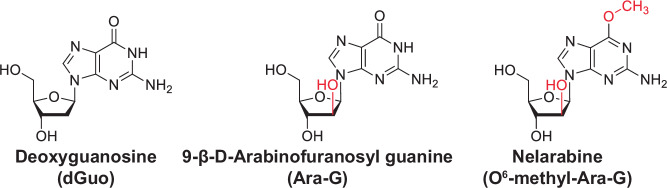
Chemical structure of nelarabine. Nelarabine is a prodrug of a 9-β-D-Arabinofuranosyl guanine (ara-G), which is an analog of the endogenous DNA precursor deoxyguanosine (dGuo). Structures of each are shown with chemical groups in red indicating modifications compared to dGuo.

In the mid-1970s it was reported that a child lacking purine nucleoside phosphorylase (PNP) activity in their red blood cells had severe T-cell immunodeficiency whilst retaining normal B-cell immunity [10]. PNP catalyzes the hydrolysis of (deoxy)inosine and (deoxy)guanosine to hypoxanthine and guanine respectively. Of these four substrates, only deoxyguanosine (dGuo) was demonstrated to be selectively toxic to PNP-deficient cells in low concentration [11, 12], indicating it could be the culprit of T-cell deficiency. The combination of this selective cytotoxicity and the observation that T-cells have a unique ability to accumulate deoxyguanosine triphosphate (dGTP) from exogenous dGuo [13], led to the hypothesis that a dGuo analog resistant to PNP hydrolysis would be beneficial in the treatment of T-cell neoplasms. Previously, in the early 1960’s, Elmer J. Reist and Leon Goodman had completed the chemical synthesis of a series of arabinosyl derivatives of the common nucleosides including the guanosine analog 9-beta-d-Arabinofuranosylguanine (ara-G) (Fig. 1) [14]. Ara-G was chosen for investigation as a treatment of T-cell neoplasms, and was subsequently shown to not be hydrolyzed by PNP [15] and was selectively toxic to T-ALL cells [15, 16]. Subsequent metabolic studies showed that T-ALL cells have a higher accumulation and retention of the ara-G triphosphate metabolite ara-GTP than other leukemic cells [17–19], which was understood to be the active metabolite. Whilst these studies were promising, a few hurdles limited the development of ara-G for clinical use. Firstly, the traditional synthesis of ara-G [14] was difficult, limiting its availability for researchers, which could be overcome with enzymatic [20] or bacterial [21] synthesis. Furthermore, ara-G has poor water solubility, which hampered in vivo studies. To overcome this, the water-soluble prodrug Nelarabine (O^6^-methyl ara-G) was developed, which is quickly converted into ara-G by adenosine deaminase (ADA) (Fig. 1) [22]. The improvement in production and solubility allowed the first clinical trial to be initiated in 1994 [23–25]. Although not the focus of the present review article, it is worth highlighting that an orthogonal approach to exploit the sensitivity of (malignant) T-cells to dGTP was the development of PNP inhibitors such as the purine analog forodesine [26].

## Overview of clinical approval and use

The first clinical trial for nelarabine, started in 1994, showed that nelarabine is quickly converted to ara-G in vivo and that the peak ara-G concentration is correlated to the administered dose [23, 24]. Reflecting previous in vitro studies, T-ALL patients accumulated higher ara-GTP concentrations than T-cell chronic lymphocytic leukemia (CLL), non-T leukemias or PBMCs from lymphoma patients [24]. Higher ara-GTP concentration was associated with complete or partial response in this phase I clinical trial [24], consistent with the in vitro studies indicating the triphosphate metabolite is the principal active metabolite. The success of two phase II trials, one in pediatric patients [27] and one in adult patients [28], lead to the approval of nelarabine for the treatment of relapsed or refractory T-ALL or T-cell lymphoma (T-LBL) by the FDA in 2005 [7]. Despite the success, neurologic adverse events ≥ grade 3 were reported in 18% of pediatric [27] and 5% of adult cases [28]. Similarly, in a larger phase II clinical trial in adults, only 7% of patients were reported with a neurologic adverse events ≥ grade 3 [29].

After the success of nelarabine as mono-treatment in salvage therapies, nelarabine was evaluated in the treatment of patients with newly diagnosed T-ALL. In pediatric patients with a slow early response to prednisolone, which is a predictor of poor outcome [30], the addition of nelarabine to the BFM-86 treatment regimen led to a 5-year event-free survival (EFS) similar to the patients with a rapid early response to prednisolone who were not treated with nelarabine, while the neurotoxicity remained acceptable [31]. Further studies showed that nelarabine can be safely added to the hyper-CVAD treatment regimen in adults [32, 33] and to the COG augmented BFM backbone in pediatric and young adult patients in a randomized setting [34]. A randomized controlled phase III study in pediatric and young adult patients on a COG augmented BFM backbone showed that nelarabine significantly improved the 5-year disease-free survival from 82.1% to 88.2%, irrespective of patient age, without an increase of toxicities [35]. Nelarabine is currently evaluated in several clinical trials, amongst others in the front-line treatment of pediatric and young-adult (1-45 years old) high-risk T-ALL patients in the European ALLTogether clinical study protocol (NCT04307576). While nelarabine seems successful in T-ALL, no difference in disease-free survival was observed in a randomized trial in children and young adults with T-LBL, while patients treated with nelarabine experienced an increase in neurological toxicities [36]. In a study including three T-cell chronic lymphocytic leukemia patients, no response to nelarabine was observed [24]. In a phase I trial, nelarabine seems successful in indolent leukemias (B-cell chronic lymphocytic leukemia and T-cell prolymphocytic leukemia). [37] For a recent review of the clinical data regarding nelarabine use in T-ALL treatment, see Shimony et al. [38] and Miller et al. [39].

## Literature annotation

As a prelude to in-depth characterization of the molecular mode-of-action of nelarabine, we performed a semi-systematic literature review. This was partly motivated by the knowledge that nelarabine remains the least studied FDA/EMA-approved anti-cancer nucleoside analog (Supplemental Fig. 1A). All available papers were collected from PubMed and evaluated for relevance based on the abstract (detailed in Supplemental Fig. 1B; Supplemental Table 1). This analysis revealed that about a third of the available literature (122 studies) contains preclinical research, and those informing upon nelarabine metabolism and mode-of-action are discussed in detail below. Additional studies that were deemed of interest during writing were also included.

## Intracellular metabolism & molecular mode-of-action

### Selectively for T-lymphoblasts

One intriguing feature of nelarabine and ara-G is their apparent selectivity for inhibiting the proliferation of T-lineage cells. As discussed above, this property underpins the development of this therapy for treatment of T-cell malignancies, and was first documented in a series of independent studies in the early 1980’s comparing dGuo and ara-G. Using incorporation of labeled DNA precursor ^3^H-thymidine as a proxy for measuring cell viability in a small collection of leukemic cell lines, both dGuo and ara-G could selectively inhibit ^3^H-thymidine incorporation, and thereby DNA synthesis, in T- over non-T leukemic cell lines [15]. In accordance, T-leukemic cells accumulated high levels of the triphosphate metabolite which was not observed in B-leukemic cells [15], and further underscoring therapeutic potential, ara-G was shown to inhibit ^3^H-thymidine incorporation into leukemic cells from three T-ALL patients ex vivo [15]. Shortly after, using B- and T-leukemic cell lines derived from the same patient, and a different experimental approach, these findings were independently validated [16]. Subsequently, it was observed that whilst phosphorylation of ara-G (i.e., activation) in another T- and B-leukemic cell line was comparable, the B-leukemic cell line degraded the triphosphate whilst the T-leukemic cell line did not, indicating slow or absent catabolism of the active metabolite in malignant T-cells may be the explanation for differential sensitivity [17]. This selective catabolism in B-leukemic cells was not observed (as strongly) with other arabinose nucleoside analogs (ara-C, F-ara-A, and ara-A), which was consistent with little/weak selectivity for these nucleosides for T- over B-leukemic cell lines when tested in a larger panel of 8 lines in total [17]. A subsequent study comparing ara-C and ara-G in different leukemia types ex vivo showed selectivity of ara-G for T-ALL over chronic myelogenous leukemia (CML) and acute myeloid leukemia (AML) which was consistent with selective accumulation of ara-GTP, indicating the results previously obtained in cell lines was representative of freshly isolated leukemic cells also [19]. Furthermore, a later study using an expanded panel of 96 diagnostic pediatric leukemia patient samples also observed selectivity for T-ALL [40]. This selectivity of ara-G for T-lineage cells has also been confirmed outside the context of leukemia treatment, for instance, in the utility of ara-G based PET probes for specifically visualizing T-cells [41–43].

Recent studies have uncovered a principal cause of differential sensitivity between T- and B-ALL cells highlighting the dNTP hydrolase SAMHD1, or rather lack thereof, as a key component. SAMHD1 is a dNTP triphosphohydrolase capable of deactivating several anti-leukemic nucleoside analogs, which was characterized extensively for ara-C therapy in AML [44, 45]. Leukemic cells with SAMHD1 expression ablated using CRISPR/Cas9 are hypersensitive to nelarabine [46] and subsequently a thorough analysis of SAMHD1 expression and ara-G/nelarabine sensitivity in ALL revealed that the lack of SAMHD1 expression in T-ALL could largely account for the differential sensitivity as compared to B-ALL [47]. This is consistent with earlier studies documenting a lack of ara-GTP catabolism in T-ALL cells [17].

**Fig. 2:**
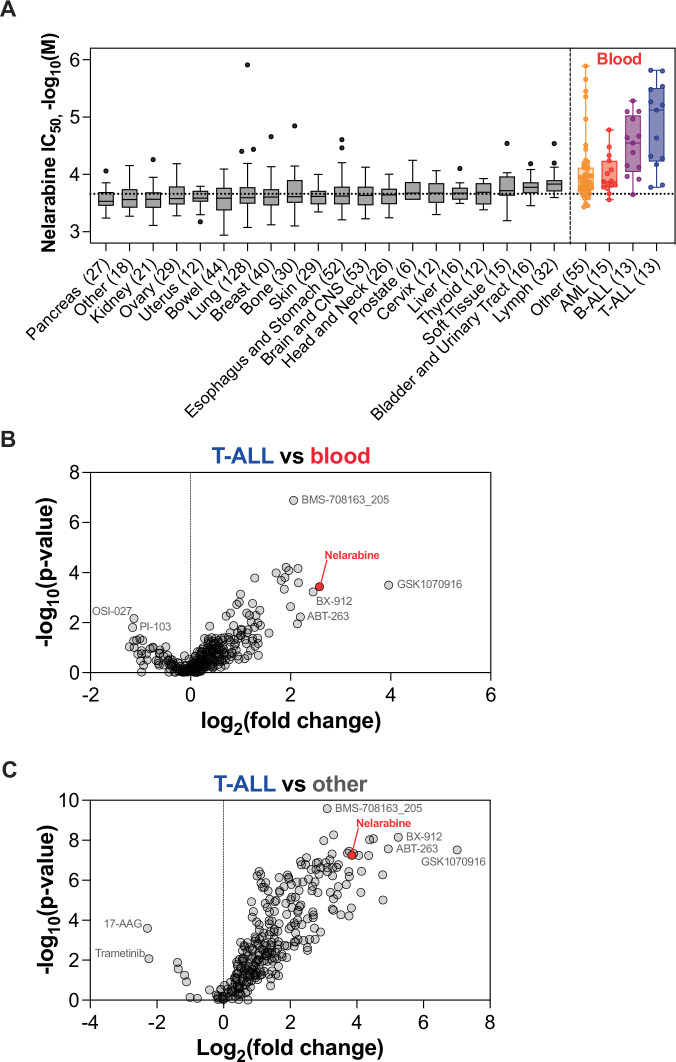
Nelarabine is selective for T-ALL cell lines. **A** Nelarabine sensitivity of all cell lines in the Genomics of Drug Sensitivity in Cancer (GDSC) database [113]. Cell lines are grouped by tissue of origin. All tissues with <6 samples were grouped in other, number in brackets shows the number of cell lines per group. Detailed annotation of all cell lines that were annotated as blood in the GDSC database (other, AML, B-ALL, and T-ALL) is done based with the cellosaurus database. Data is shown as -log_10_(IC_50_ of nelarabine in M), box shows the median and 25-75 quartile, and whiskers are plotted according to the Tukey method, outliers are shown as dots, with the exception of the blood cell lines, in which all datapoints are shown and the whiskers represent minimum to maximum value. The dotted line shows the median of the whole dataset. Nelarabine sensitivity does not depend on p53 mutation status (Supplemental Fig. 2). **B**, **C** Selectivity of nelarabine for T-ALL within all cell lines (**B**) or only blood cell lines (**C**) compared to other drugs in the GDSC database. Red dot shows nelarabine, grey dots show other drugs (*n* = 295). P-value is calculated using the two-sided Mann-Whitney U-test on the −log_10_(IC_50_) values. Log_2_(fold change) is calculated as log_2_(median IC_50_ T-ALL / median IC_50_ other cell lines or blood cell lines). Data represented in these plots can be found in Supplemental Table 2.

With the availability of large scale pharmacogenomic screens in cancer cell lines, the selectivity of nelarabine for T-ALL becomes even more clear (Fig. 2A). Most T-ALL cell lines are sensitive to nelarabine, as well as many B-ALL cell lines and a handful of blood cell lines, while AML cell lines are not sensitive to nelarabine. Only the occasional non-blood cell line shows sensitivity, such as the lung cell line NCI-H1417, which is also the most sensitive cell line in this dataset. Nelarabine is also amongst the most selective drugs for T-ALL when evaluated in all cell lines or restricted to only blood cell lines (Fig. 2B, C).

### Metabolism—activation and deactivation

**Fig. 3:**
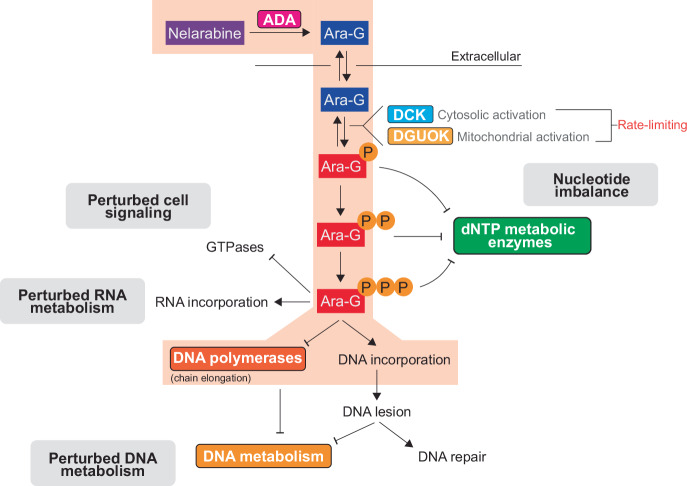
Metabolism and potential cellular targets of nelarabine. Nelarabine is converted to ara-G via adenosine deaminase (ADA). Once inside cells, ara-G can be phosphorylated by deoxycytidine kinase (DCK) in the cytosol or deoxyguanosine kinase (DGUOK) in the mitochondria before further phosphorylation events catalyzed by nucleotide kinases. Phosphorylated metabolites could perturb deoxynucleotide pools via inhibition or modulation of deoxynucleotide metabolizing enzymes. The principal active metabolite, ara-GTP, could potentially perturb GTPase function in addition to being incorporated into nascent RNA and perturbing RNA metabolism. Incorporation into DNA, particularly nuclear DNA, is a principal mode of cytotoxicity. Here, ara-GTP incorporation can inhibit the DNA synthetic reaction and/or perturb downstream DNA metabolic processes. Highlighted in red is the main mode of action discussed in the literature. Figure adapted from [114].

Nelarabine, like all nucleoside analogs, is administered as an inactive pro-drug and uses the cells endogenous metabolic pathways to be converted into its activated form (Fig. 3). Demethoxylation is required first, converting nelarabine to ara-G, and is catalyzed by the enzyme ADA [22]. This process occurs quickly after administration to patients, with 94% of nelarabine converted to ara-G after 1 hour and a longer half-life of ara-G than nelarabine [23, 24]. Ara-G is then taken up by cells using ENT1 and a second method, likely to be ENT2 [48]. In experiments in vitro, this transport leads to equal ara-G concentrations in the cytosol and mitochondrial compartments as is supplied in the culture media [49]. While ara-G was initially described as a PNP resistant dGuo analog, data suggests that PNP can still breakdown ara-G with a low efficiency [22, 49]. Although low, it might be of biological relevance, as some tissues such as erythrocytes express high levels of PNP.

After intracellular uptake, ara-G is then converted to ara-G monophosphate (ara-GMP), diphosphate (ara-GDP), and its active triphosphate metabolite ara-GTP. The rate-limiting step in this activation is the first phosphorylation step towards ara-GMP [50]. For this activation, ara-G uses the endogenous salvage pathway enzymes deoxyguanosine kinase (DGUOK) in the mitochondria and deoxycytidine kinase (DCK) in the cytosol. Both enzymes can convert ara-G to ara-GMP but with different efficiencies [51–55]. DGUOK phosphorylates ara-G with a similar efficiency as its endogenous counterpart dGuo, whilst this efficiency is less for DCK [51, 52]. DGUOK prefers ara-G as a substrate over dGuo in pH >7 [53]. Due to these differences, ara-G conversion is more efficient in mitochondrial extracts than in cytosolic extracts [51], but DCK is capable of catalyzing this reaction and is the preferred enzyme at high ara-G concentrations [49, 54]. The concentration of ara-G phosphorylated metabolites is higher in mitochondria [49]. Due to differences in DCK and DGUOK expression in crude cell extracts, less than 50% of ara-G is converted by DGUOK [52]. Similarly, blocking DCK with dCyd reduces intracellular ara-G phosphorylated metabolite concentrations and protects cells from ara-G related cytotoxicity [15, 49, 55]. However, overexpression of both DCK and DGUOK sensitizes cells to ara-G induced cytotoxicity [50], supporting that both salvage kinases can contribute to activation of ara-G.

As nelarabine and ara-G are dependent on activation by endogenous metabolic enzymes, expression of these enzymes can correlate to ara-G toxicity. For instance, ENT1 and ENT2, the transporters responsible for the cellular uptake of ara-G, mRNA expression is higher in primary patient samples that are sensitive to ara-G [40]. Another study evaluated the expression of several metabolic enzymes and observed a correlation between the (ENT1 × DCK)/(CDA × RRM1) ratio and ara-G cytotoxicity in ex vivo primary T-ALL samples [56]. Following the same rationale, a correlation of the ENT1 + DCK expression and ara-G cytotoxicity in cultured cell lines has also been reported [57]. While activation of ara-G is related to the cytotoxicity, inactivating enzymes can influence the efficacy as well. The expression of dNTP hydrolase SAMHD1, which is typically not expressed in T-ALL [47], is negatively correlated with nelarabine cytotoxicity and ablation of SAMHD1 expression sensitizes cells to nelarabine [46, 47]. NT5C2, a nucleotidase, can potentially inactivate ara-GMP to ara-G, but no enzymatic proof has been published to our knowledge. Circumstantial data suggests that it might be involved, as NT5C2 targeting guides were depleted by nelarabine treatment in a CRISPR screen [58]. However, the expression of relapse associated NT5C2 mutations, associated with resistance to thiopurines antimetabolites, do not alter the nelarabine cytotoxicity [59].

### Targeting the nuclear genome

**Fig. 4:**
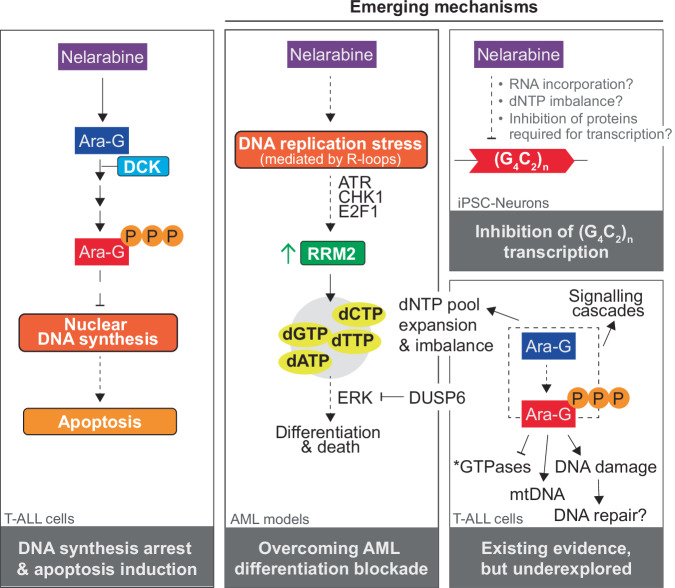
Established and emerging molecular mechanisms of nelarabine. Early studies in T-ALL cells established the current molecular mechanism of nelarabine, which involved deoxycytidine kinase (DCK) mediated activation to produce the triphosphate metabolite ara-GTP which can arrest nuclear DNA synthesis inducing apoptosis. Dashed arrows indicate potential knowledge gaps. Recent studies have highlighted additional emerging molecular mechanisms of nelarabine. These include inducing differentiation and cell death in AML models, mediated through a multistep process involving DNA replication stress signaling, upregulation of ribonucleotide reductase (RNR) subunit RRM2 promoting dNTP pool imbalance, and ERK signaling, which can be counteracted by DUSP6 [58]. In iPSC-Neurons, nelarabine was demonstrated to inhibit transcription of (G_4_C_2_)_n_ repeats by an undefined mechanism [65]. Additionally, we highlight mechanisms for which evidence exists in T-ALL cell models but have been little explored. Details in the main text. Asterisk indicates evidence for GTPase inhibition does not exist in T-ALL cells but rather in biochemical experiments [101, 102].

The principal target of many anti-cancer nucleoside analogs is understood to be nuclear DNA [9], and some of the first experimental data collected from ara-G exposed cells supported this mode-of-action for this analog too (Figs. 3, 4). Early studies used ^3^H-thymidine incorporation into DNA as a proxy for measuring viable cells and thereby demonstrated ara-G inhibited DNA synthesis in leukemic cells in a dose-dependent manner [15, 18, 60]. Later, ara-G incorporation into DNA was demonstrated to be a necessary step for apoptosis induction in the T-lymphoblastic line CCRF-CEM [61]. Using chemical blockers of cell cycle progress (double thymidine block or DNA polymerase inhibitor aphidicolin), the authors showed that whilst accumulation of ara-GTP was independent of cell cycle phase, the incorporation of ara-GTP into DNA and the subsequent induction of apoptosis was dependent upon S-phase entry [61]. Accumulation of cytotoxic levels of ara-GTP outside of S-phase did not result in apoptosis induction. The study also demonstrated that ara-GTP incorporation into cellular DNA resulted in DNA synthesis inhibition [61]. These data are in accordance with biochemical primer-extension assays with several DNA polymerases from different sources [CHO cells [62], murine cells [63], and human cells [22]] that showed the use of ara-GTP in the DNA synthetic reaction, utilized in place of dGTP, was inhibitory to primer elongation [22, 62, 63]. Inspection of the reaction at single nucleotide resolution indicated that DNA synthesis (at least by Pol α from CHO cells) was arrested upon ara-GTP incorporation into the chain (i.e., chain termination) [62]. Of the DNA polymerases tested (Pols alpha, beta, gamma, delta and epsilon), Pol α, the proofreading-deficient DNA polymerase responsible for initiating DNA synthesis, was the most sensitive to inhibition by ara-GTP [22, 63]. Although chain-termination was observed in biochemical polymerase assays, ara-GTP is not strictly a chain terminator as this analog contains the 3’-OH moiety required for DNA synthesis by polymerases, unlike actual chain terminating di-deoxynucleotides. Consistent with this, analysis of ara-GMP molecules present in DNA following ara-G treatment of leukemic cells revealed the majority of this analog was incorporated at internal sites in the DNA molecule, rather than at termini, consistent with cellular polymerases successfully extending from ara-GMP containing DNA molecules [64]. In summary, although the DNA molecule is clearly an important target for ara-G/nelarabine, our understanding of the DNA synthetic process, both the biochemistry on isolated proteins but also in a cellular context, has greatly advanced since these studies, and a focused re-analysis of how ara-GTP perturbs DNA synthesis could yield important insights. For instance, knowledge of how ara-GMP containing DNA templates are replicated is lacking together with a broader assessment of the DNA polymerase families, as there are currently 16 known enzymes and many of these are inherently more flexible than the cellular replicases previously tested.

Two recent studies have shed additional light upon how ara-G/nelarabine can perturb nucleic acid metabolism (Fig. 4). Nelarabine was identified as a hit in a phenotypic screen in patient-derived induced pluripotent stem-cell-derived neurons for inhibitors of dipeptide repeat expression, which is of relevance to amyotrophic lateral sclerosis and frontotemporal dementia [65]. Nelarabine, together with the other nucleoside analog hits decitabine and entecavir, were shown to specifically inhibit transcription of the (G_4_C_2_)n sequence encoding the dipeptide. Although the mechanism was not established, the authors suggested three models consistent with their data, including a preference for incorporation of the triphosphate into these nascent transcripts resulting in degradation, perturbation of nucleotide pools possibly via ribonucleotide reductase (RNR), or that the analogs directly inhibit DNA/RNA helicases (or other proteins) required for transcription of these repeat sequences [65]. This study also contains RNA-seq analysis of nelarabine-treated post-mitotic neurons, in which the authors note negligible toxicity, and identify 64 differentially expressed genes including ribosomal components and p53 target genes [65].

The second study stems from an in silico and subsequent phenotypic screen to identify compounds that overcome AML differentiation blockade, which identified and validated nelarabine as one such compound [58]. Following extensive characterization of nelarabine-exposed AML cells, the authors compile a model in which nelarabine treatment induces R-loop (RNA:DNA hybrid) mediated replication stress activating ATR-Chk1 signaling causing upregulation of RRM2, the small subunit of RNR. This upregulation results in dNTP pool expansion and imbalance, with the dGTP pool becoming the largest, which ultimately results in elevated ERK signaling that drives differentiation [58]. To identify possible drugs to combine with nelarabine in this context, the authors also conducted a whole genome CRISPR screen in an AML cell model with acquired resistance to nelarabine, using nelarabine treatment as phenotypic selection in the screen, and identified DUSP6 (dual-specificity phosphatase 6) as one hit. The authors show that depletion or pharmacological inhibition of DUSP6 potentiates nelarabine-induced ERK activation, differentiation, and apoptosis [58]. Of note, ERK activation has been documented in T-ALL following nelarabine treatment [66].

### Targeting the mitochondria

Given the higher activity of DGUOK over DCK in phosphorylating ara-G [51, 52], and the exclusive localization of this enzyme to the mitochondria, several studies have interrogated the involvement of mitochondria and mitochondrial DNA (mtDNA) in the cytotoxicity of ara-G. Experiments in which T-lymphoblast cells are exposed to radiolabeled ara-G followed by in situ visualization of autoradiography clearly show a punctate cytosolic pattern (in addition to nuclear signal), which is consistent with incorporation into mtDNA, and distinct from ara-C [67]. Furthermore, DCK KO CHO cells retain this ara-G mitochondrial staining, in contrast to ara-C nuclear signal which is lost, which would be consistent with DGUOK activation [67]. Supporting this, DGUOK knockdown cells show decreased incorporation of ara-G into mtDNA [68]. Despite incorporation, a study investigating effects of ara-G on mtDNA content and function revealed no defects [69], although an important note here is that sub-toxic concentrations of ara-G (10-fold lower than IC_50_ values) were used when assessing mitochondrial phenotypes, which could be too low. However, at least in MOLT-4 T-ALL cells, mtDNA is not required for acute cytotoxicity, as Rho0 cells lacking mtDNA could still be killed by ara-G treatment, although curiously with a 10-fold increase in sensitivity [70]. This is in line with another study that generated a cell model with decreased mtDNA content and identified enhanced sensitivity to ara-G [71]. This suggests use of ara-GTP in mtDNA replication could protect cells from acute cytotoxicity from this metabolite by limiting its availability for nuclear DNA synthesis. A recent study that investigated the utility of nelarabine to overcome differentiation blockade in AML evaluated mitochondrial morphology in an AML cell line exposed to nelarabine with electron microscopy and observed disruption of mitochondrial matrix morphology and loss of mitochondrial cristae [58], demonstrating nelarabine can negatively impact this organelle. The underlying mechanism was unclear but induction of ROS was suggested to be involved [58].

### Disruption of dNTP homeostasis

While nelarabine requires the cells endogenous metabolic pathways to be activated into its active components, it can also potentially interfere with these pathways (Figs. 3, 4). At high concentrations, ara-G is a competitive inhibitor of PNP in biochemical assays [72, 73]. Nelarabine, but not ara-G, can inhibit the ENT1 transporter, and to a much lesser extent the ENT2 transporter in cell lines overexpressing the relevant transporters [74]. Ara-G is activated by DGUOK, but can also act as a competitive inhibitor in biochemical assays [53, 75]. As dGuo is an inhibitor of RNR, it is suggested that ara-G is an inhibitor of this enzyme also [49]. Inhibition of RNR with dGuo results in an increase in dATP and dGTP concentration, coupled with reduced dTTP and dCTP concentrations [49]. However, treatment with ara-G results in expansion of dNTPs, suggesting that it is likely not an inhibitor of this enzyme [49, 58]. This expansion follows a DNA replication stop, so it could be the result of reduced dNTP use in newly synthesized DNA [49]. On the other hand, the expression of RRM2 is increased upon treatment with ara-G [58]. As the dNTP expansion is prevented with an RNR inhibitor or RRM2 knockdown, it is likely that the increased RRM2 expression as a result of ara-G treatment is responsible for the dNTP expansion [58]. This overexpression of RRM2 could be a result of ara-G-induced DNA damage [58].

### Lessons from cell lines with acquired resistance

The activation or mode-of-action of a drug can be explored by evaluating resistance mechanisms to the specific drug. This has been applied in nelarabine research by producing nelarabine/ara-G resistant cell lines. This is usually done by prolonged culturing in the presence of increasing concentrations of ara-G, which has been done by several authors, namely Shewach et al. (using Molt-4) [18], Curbo et al. (using CCRF-CEM) [76], Lotfi et al. (using MOLT-4) [77–80], Yamauchi et al. (using CCRF-CEM) [81, 82], and Yoshida et al. (using B-ALL cell lines BALL and Nalm-6, and T-ALL cell lines MOLT-4 and SKW3) [83].

These studies revealed that cell lines can become resistant via different mechanisms (Fig. 6), which can also occur sequentially by continued selection using increasing ara-G concentration [76]. A common mechanism of resistance is the reduced accumulation of ara-GTP [18, 77, 81]. Often, this is due to a reduced expression or activity of DCK [76, 77, 81, 83] and/or DGUOK [77, 81]. In most instances, uptake of ara-G remains stable [76, 77], however, reduced ENT1 expression, which suggests reduced ara-G uptake, was observed as well [81]. The reduced expression of DCK was not due to a change in methylation of the *DCK* promotor but is due to decreased acetylation of histone H3 and H4 at the *DCK* promotor [83]. Treatment with the histone deacetylase (HDAC) inhibitor vorinostat reversed the ara-G resistant phenotype in all four cell lines and restored DCK expression [83].

The reduced ara-G phosphorylation is associated with less incorporation of ara-G into mitochondrial [76, 81] and nuclear [81] DNA. The reduction of ara-G incorporation into DNA was similar between the nuclear and mitochondrial compartments [81]. Wildtype cells with depleted mtDNA remained sensitive to ara-G, suggesting that the incorporation of ara-G into mtDNA is not the mechanism of cytotoxicity [81], consistent with other studies using mtDNA-depleted cells [69, 70]. Ara-G resistant cells had a lower potential for apoptosis, showing a lower membrane potential than their wildtype counterparts [78], as well as reduced expression of proapoptotic BCL2 [78], BAX [81], and BAD [81] and increased expression of antiapoptotic BCL2L1 [78, 81]. In addition, anti-FAS antibody treatment did not induce apoptosis, while it was able to induce apoptosis in wildtype cells [78]. Ara-G resistant MOLT-4 cells were also resistant to vincristine and daunorubicin [77]. These cross-resistant cells showed increased expression of the protein p-gp and its encoding gene *ABCB1* [77, 79]. Microarray analysis of the resistant cells revealed that several fetal hemoglobin genes were upregulated in the ara-G resistant cells [79]. Expression of ABCB1 and fetal hemoglobin genes are associated with hypomethylation, and indeed, ara-G was shown to reduce global methylation levels [79, 80]. Inhibition of p-gp with SB203580 resulted in partial reversion of the resistance to ara-G [80]. As ara-G is not a substrate for p-gp, and fetal hemoglobin or its breakdown products can be toxic at high concentrations, the authors suggest that the increase in p-gp expression is induced to export fetal hemoglobin [79, 80], meaning that increased fetal hemoglobin expression might be the reason that these cells are resistant to ara-G.

Resistance to ara-G can result in cross-resistance to other nucleoside analogs, such as ara-C, ara-T, dGuo, dF-dC, dF-dG, CdA, and 2-F-ara-A [18, 76, 77, 81, 83]. Interestingly, this cross-resistance differs per developed cell line. For example, Curbo et al. report increased resistance towards dF-dC [76], while Lotfi et al. observed increased sensitivity to dF-dC in their ara-G resistance clones [77]. Both studies indicated a reduction in DCK activity, while DGUOK activity was only reduced in the study by Lotfi et al. Furthermore, Lotfi et al. report an increase in TK2 activity, which can phosphorylate dF-dC and might thereby increase the sensitivity to this drug [77]. Curbo et al. developed two independent ara-G resistant clones [76]. While both clones showed resistance to ara-C, dF-dC, and dF-dG, the resistance of clone 2 was much more pronounced. Their data suggested that this clone is deficient in DCK activity, while this is not affected in clone 1 [76]. In the study of Shewach et al., ara-G resistant MOLT-4 cells were cross-resistant to dGuo, the endogenous counterpart of ara-G [18]. In contrast, ara-G resistant CCRF-CEM cells were not cross-resistant to dGuo in the study of Yamauchi et al. [82].

### Lessons from preclinical drug combinations

Insights into the molecular mechanism of a drug can be gleaned from how this drug is combined with other therapeutics. T-ALL patients are often treated with a plethora of drugs and nelarabine is used in combination in clinical trials and in clinical practice (reviewed previously [38, 39]). However, delineating mechanistic insights from clinical combinations is difficult, and thus here we focus upon preclinical research. Surprisingly, there are relatively few studies on drug combinations with nelarabine.

As described above, DCK and DGUOK activate ara-G. Therefore, increased activity of these enzymes could increase intracellular ara-GTP concentrations and thereby potentiate nelarabine treatment. RNR inhibitors reduce intracellular dNTP concentrations, and as dCTP and dGTP, which feedback inhibit DCK and DGUOK respectively, thus a reduction in concentration of these dNTPs could alleviate the inhibition on these enzymes and thereby potentiate ara-G treatment. Pretreatment with the RNR inhibitor dF-dC results in reduced intracellular dCTP, dATP, and dGTP concentrations, and as hypothesized by Gandhi and Plunkett, this results in increased phosphorylation of ara-G, resulting in higher intracellular concentrations of ara-GTP [84]. In line with these results, the combination of ara-G and the RNR inhibitor fludarabine, which can reduce intracellular concentrations of dCTP and dGTP, increases the intracellular ara-GTP concentration in an ex vivo setting [85]. Based on this, a clinical trial combining fludarabine with nelarabine was initiated and subsequently published in 2001 [86]. In this pilot protocol, patients received a combination of fludarabine and nelarabine, which was well tolerated, but as all patients received the combination, efficacy over monotherapy cannot be established [86]. A later trial, published in 2020, combining fludarabine, nelarabine, and etoposide (a topoisomerase II inhibitor) concluded that this combination was safe, but the trial was terminated early due to recruitment difficulties [87]. To our knowledge, the combination of fludarabine and nelarabine has not been explored further in preclinical models.

Intriguingly, ara-G treatment was reported to augment DCK protein abundance, shown in the APL model HL-60, and thereby ara-G was used in combination with ara-C to increase activation and cytotoxicity of this deoxycytidine analog [88], but the underpinning mechanism was not described. This study concluded with a triple combination of ara-G, ara-C, and a Bcl-2 inhibitor (YC137), which significantly increased apoptosis induction in HL-60 cells over the ara-C and YC137 combination (unfortunately, the ara-G and YC137 combination was not evaluated) [88]. Modulation of apoptosis is a promising basis of modern drug combinations that have been little explored with nelarabine. Another exception is nitric oxide (NO) donating drugs, known to induce apoptosis, and a synergistic interaction was reported between nelarabine and DETA-NO in CLL models [89].

Evaluation of a panel of T-ALL cell lines showed those with relative resistance to nelarabine had increased activation of AKT and MEK/ERK by nelarabine treatment, while this is not the case for sensitive cell lines [66]. The pan PI3K inhibitor ZSTK-474 was synergistic with nelarabine in these resistant cell lines, as well as two ex vivo T-ALL patient samples [66]. ERK activation following nelarabine treatment has also been reported in AML cell models [58]. Here, pharmacological inhibition of DUSP6, an ERK-specific phosphatase, was shown to potentiate nelarabine anti-leukemic activity in a xenograft mouse model [58].

Nelarabine and ara-G target DNA, as described above, which means that a mechanism-based combination could include other DNA targeting agents. The addition of aphidicolin, a DNA polymerase α and δ inhibitor, to ara-G treatment, increases the percentage of apoptosis in HL-60 cells, as well as reduces cell growth [90]. However, the effects of this combination may be dose-dependent given that earlier studies established active DNA synthesis is important for ara-G cell killing [61], thus inhibition of DNA synthesis would reduce incorporation of ara-GTP and could be antagonistic. One promising combination is with the prodrug OBI-3424 that is converted to a potent DNA-alkylating agent by AKR1C3, which is higher expressed in T-ALL and therefore this preclinical drug is more active in T-ALL [91]. This drug, and the follow-up drug ACHM-025, in combination with nelarabine is potent in in vivo models [91, 92].

### Neurotoxicity

The dose-limiting toxicity of nelarabine is neurotoxicity [25], which can be managed with dosing on alternate days (1500 mg/m^2^/day on day 1, 3, and 5) [28, 29] or daily dosing with lower doses (650 mg/m^2^/day for 5 consecutive days) [32–35]. Ara-G can cross the blood-brain barrier, resulting in a cerebrospinal fluid (CSF) ara-G concentration of 23% of the plasma concentration in primates [93]. This penetration into the CFS could be the reason that less CNS relapses were seen in a phase III trial with nelarabine added to a COG augmented BFM backbone [35]. The molecular basis of nelarabine-induced neurotoxicity has not been investigated preclinically. CNS and brain cancer cell lines are not more sensitive to nelarabine compared to those derived from other solid tumor types (Fig. 2A). A recent study observed no toxicity in post-mitotic neurons at 10 µM nelarabine based on total protein levels, tubulin staining, and live/dead staining [65]. These data, although minimal, might suggest that neurotoxicity might not be due to cell death, but rather an altered cell function. It has been speculated that ara-GTP could interfere with GTP-dependent motor proteins and disrupt intracellular transport in axons leading to loss of neuronal function [7], but this is yet to be investigated. A thorough molecular understanding—defining which nelarabine metabolite is responsible and the precise mechanism – is critical and may open avenues to separate efficacy and toxicity. Neurotoxicity is not restricted to ara-G treatment, but other T-ALL chemotherapies, such as vincristine, L-asparaginase, methotrexate, and cytarabine can cause neurotoxicity as well [94].

## Conclusions & future directions

Nelarabine is becoming increasingly important in T-ALL therapy [38]. Yet our understanding of the molecular underpinnings of how this drug works is derived from surprisingly few studies. Furthermore, these studies, because of when they were conducted, whether in the late 1980’s or early 2000’s, are often of limited scope, especially in comparison to the present ideal of pre-clinical drug target validation studies [95]. A clear understanding of a drugs molecular mechanism(s) is a critical step to using these therapies with increased precision, facilitating mechanism-based decisions in the clinic [8]. It is with this goal that we sought to semi-systematically review the available preclinical literature on nelarabine, to understand what is known and unknown, and to serve as a prelude to future in-depth mechanistic studies by the research community.

Accumulation of nelarabine active metabolite(s), principally the triphosphate metabolite ara-GTP, is clearly critical to efficacy. This is one area of research in which we have a detailed understanding of several key enzymes involved, including those controlling activation (i.e., DCK, and to a lesser, unclear extent, DGUOK) and potentially deactivation (i.e., SAMHD1 and possibly NT5C2). However, regarding the catabolic role of SAMHD1 in hydrolyzing ara-GTP, the clinical relevance of this remains unclear (given the apparent low expression of this enzyme in T-ALL) together with limited biochemical characterization of this process. Whether additional detoxification mechanisms exist for ara-G phosphorylated metabolites is also unclear, given the abundance of nucleotide hydrolases in cells [96], of which very few have been directly tested [97]. A thorough understanding of ara-G metabolic enzymes provides a foundation for further biomarker validation studies. In addition, an understanding of how single nucleotide polymorphisms (SNPs) in ara-G pathway genes impact patient response would be an important avenue of future research. This could also allow the development of polygenic nelarabine response scores to personalize therapy, akin to promising efforts in AML with ara-C [98]. An understanding of activation and deactivation enzymes could also open avenues for pharmacological control of ara-GTP accumulation, whether via activation of DCK or potential inhibition of SAMHD1 (if clinically relevant), either via small molecules or drug repurposing approaches [99, 100].

Whilst we have a detailed picture of how ara-GTP accumulates in cells, the subsequent mechanism of how this metabolite perturbs T-lymphocyte biology is less clear. A common feature of nucleoside analog drugs is their targeting of dNTP and nucleic acid metabolism, with for many, the DNA molecule being a key target [9], and it is clear this is also true of ara-GTP. Whilst actively replicating DNA is an important target, it remains unclear how ara-GTP incorporation leads to DNA synthesis arrest, as well as the resulting DNA lesions. Likewise, the specific DNA repair pathways required to tolerate or remove ara-G induced lesions in DNA are also unclear. This knowledge could be critical in efforts to identify patients most likely to respond, given that DNA repair defects are common in cancer these could be exploited to tailor therapy, and to the rational design of combinations with DNA repair inhibitors and/or DNA damaging drugs.

Are there other (protein) targets of ara-GTP, or other ara-G metabolites, outside of the DNA molecule contributing to efficacy and/or toxicity? This is a question to which we currently have little answers. There is an abundance of GTPases and GTP-binding proteins present in cells, and whether the presence of an arabinose-GTP could substitute for the endogenous counterpart and thereby perturb some cellular processes is unclear. Biochemical evidence exists for the GTPase tubulin [101, 102] and a correlation between nelarabine sensitivity and GTP-binding protein ARL4C expression was found [103], but outside of these examples, there is very little data. The impact of ara-GTP on RNA metabolism is also unclear, despite some evidence that it could target this process [65]. A thorough understanding of the role mitochondria and mtDNA play in ara-G efficacy and toxicity is also unclear. Overall, given the breadth of knowledge gaps regarding this therapy, the use of unbiased approaches, such as genome-wide CRISPR-knockout screens to identify genetic factors dictating drug action, could be a powerful way to progress the research field whilst embracing the poly-pharmacology typically associated with this class of therapy.

Nelarabine is currently combined with several therapies in clinical trials [38, 39] but a mechanistic understanding of clinical combinations [32–35] is important and severely lacking. This is critical given a key factor in the design of drug combinations is the selection of effective therapies with non-overlapping resistance mechanisms [104], and currently, informed decisions on this basis cannot be made for nelarabine. Thus, a priority would be to comprehensively define nelarabine resistance mechanisms in T-ALL, moving beyond the study of cell clones with acquired resistance and employing approaches such as functional genomics, and comparing these mechanisms with that of standard-of-care (T-)ALL therapies [105]. Furthermore, evaluating the interaction and potential synergy/antagonism between nelarabine and these standard therapies could be an additional important component of designing nelarabine combinations within current T-ALL chemotherapy regimens.

**Fig. 5:**
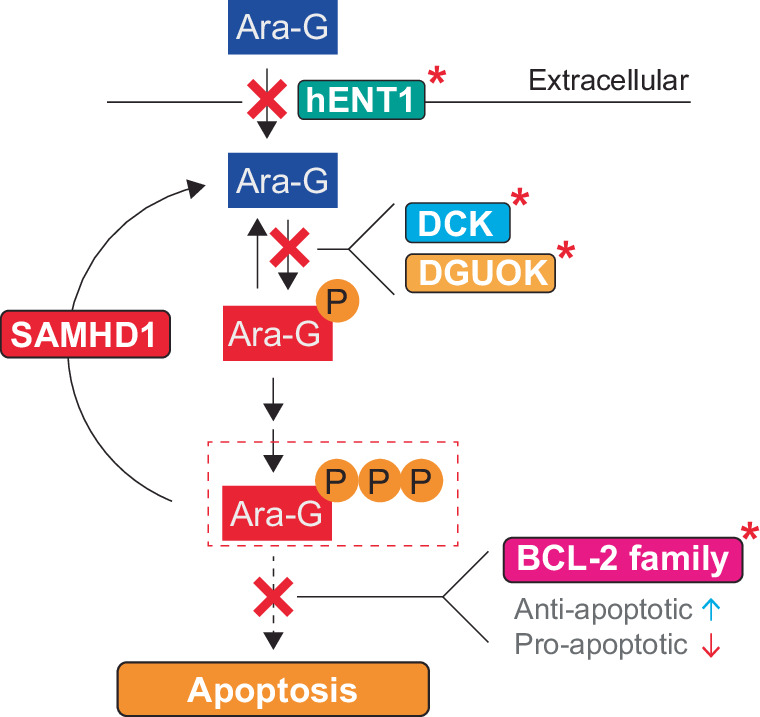
Nelarabine resistance mechanisms. Schematic detailing ara-G transport and metabolism to accumulate ara-GTP together with resistance mechanisms identified through prolonged culturing of T-ALL cells with increasing concentrations of ara-G (red asterisks). These include downregulation of ENT1, reduced expression or activity of deoxycytidine kinase (DCK) and deoxyguanosine kinase (DGOUK), or modulation of BCL-2 family members to resist apoptotic cell death.

When looking to nelarabine combinations with strong pre-clinical evidence yet to enter clinical testing, the AKR1C3-activated prodrugs OBI-3424 and ACHM-025 look particularly promising both as monotherapies and nelarabine combinations in T-ALL models [91, 92], and OBI-3424 is current under Phase II testing in R/R T-ALL (NCT04315324). If this trial proves successful, a clinical trial combining this agent with nelarabine would be an interesting next step. Modulation of apoptosis is a promising basis of modern drug combinations and evidence exists supporting its relevance for nelarabine response in T-ALL [78, 81] (Fig. 5). In our analysis of T-ALL specific drugs (Fig. 2B), the BH3-mimetic navitoclax (ABT-263) was identified, which is an effective monotherapy in T-ALL models [106]. Notably, a recent clinical study reported addition of the BCL-2 specific BH3-mimetic venetoclax to a nelarabine-containing chemotherapy regimen in T-ALL/LBL with promising results [107]. Future efforts should evaluate BH3-mimetics and other apoptosis modulators in combination with nelarabine in T-ALL both preclinically and clinically.

Manipulating nucleotide metabolic pathways to control ara-GTP accumulation is also a potential area of interest when rationally designing nelarabine combination therapies. Future efforts could build upon promising work using pre-treatment with RNR inhibitors as one approach [84, 85]. However, a recent study observed knockdown of RRM2 reverted nelarabine cytotoxicity in AML models [58], indicating further investigation may be required to delineate the precise role of RNR in nelarabine drug action. Nevertheless, future use of RNR inhibitors (or other nucleotide metabolism inhibitors) to augment nelarabine activation should prioritize non-nucleoside drugs to avoid potential cross-resistance to nelarabine via loss of nucleoside importers or DCK-mediated activation. An unexplored target in nucleotide metabolism of potential relevance is inosine-5′-monophosphate dehydrogenase (IMPDH), which catalyzes the rate-limiting step in guanine nucleotide biosynthesis. IMPDH inhibitors are emerging anti-leukemic agents particularly in the context of ALL patients resistant to frontline thiopurines owing to an activating mutation in NT5C2 [108]. Inhibition of IMPDH would deplete leukemic cells of guanine nucleotides which could compete with ara-GTP in metabolic transactions, and thus in theory, could enhance nelarabine efficacy and be of particular utility in R/R T-ALL with activating NT5C2 mutations. This has yet to be evaluated. While rational design of combination strategies based on available literature could result in promising combinations, a screening strategy where nelarabine/ara-G is combined with (pre)clinically available inhibitors could indicate the most promising combinations and provide mechanistic insight.

T-ALL currently consists of 15 known genetic subtypes [6], but it is unknown whether some of these subtypes are more sensitive or insensitive to nelarabine. Some of these subtypes might not be represented by currently available cell lines, making evaluation of nelarabine efficacy for that specific subtype troublesome. Furthermore, as T-ALL subtype representation is dependent on the age of the patient [6], nelarabine efficacy could also be dependent on age. For example, the STAG2/LMO2 subtype occurs in very young T-ALL patients (≤ 2-year-old) [6, 109] and renders the cells more sensitive to PARP inhibition, but potentially nelarabine as well [109]. A broad nelarabine/ara-G efficacy screen, ideally involving patient material to capture subtypes underrepresented in the available cell line pool, could suggest which T-ALL subtypes might benefit more or not at all from nelarabine treatment. Another approach could be to mimic putative T-ALL drivers in vitro to evaluate their effect on nelarabine efficacy.

Immunotherapies are an emerging class of therapies in the treatment of B-ALL, with FDA-approved therapies such as CAR-T cells [110]. The development of CAR-T cells directed against T-ALL cells comes with challenges, such as fratricide, T-cell aplasia or contamination of CAR-T products with T-ALL cells [111, 112]. There are currently no FDA/EMA-approved immunotherapies for T-ALL. Since the immunotherapy options for T-ALL patients are limited, nelarabine will fulfill an important role in the treatment of these patients, underscoring the need of understanding the mechanism of action of this drug.

In summary, despite the increasing importance of nelarabine in T-ALL treatment, many gaps remain in our understanding of how this drug works. If we are to use this effective medicine with more precision, it is vital that efforts are placed on deepening our molecular understanding of this anti-leukemic therapy, and we hope this review can provide a foundation for such studies.

## Supplementary information

Supplementary Figures 1-2

Supplemental Table 1

Supplemental Table 2

## Data Availability

All data analyzed during this study are included in this published article and its supplementary files.
