# Supplementary material for: Nelarabine in T-cell acute lymphoblastic leukemia: intracellular metabolism and molecular mode-of-action
Source: Leukemia. 2025 Feb 17;39(3):531–42. doi: 10.1038/s41375-025-02529-2 (PMC11879874; doi:10.1038/s41375-025-02529-2)
Supplement: Supplementary file 1 — Supplementary Figures 1-2 [file 41375_2025_2529_MOESM1_ESM.pdf]

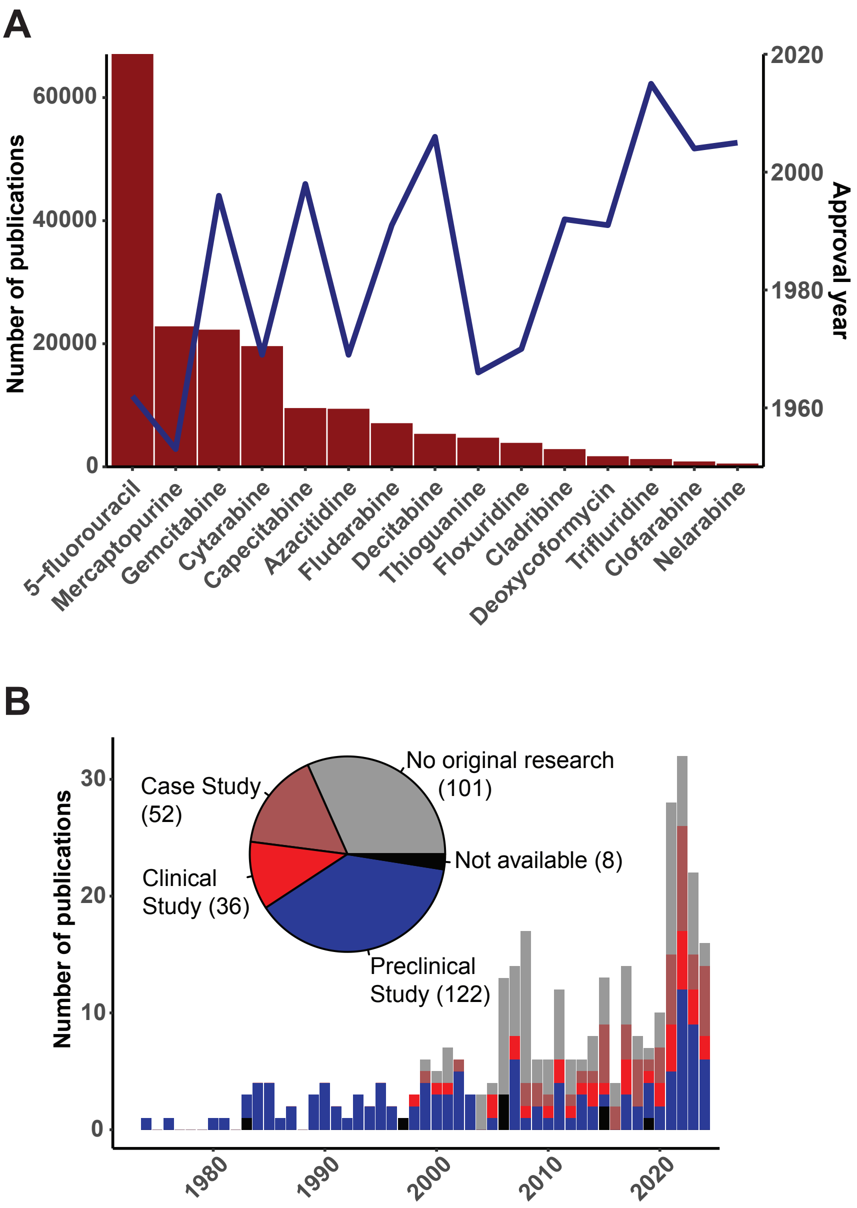

Supplemental Fig 1. Nelarabine literature overview.

A) The number of papers for each clinically used nucleoside analogue was extracted using the R package easyPubMed 2.13, using the noted nucleoside analogue as search term, with the exception of nelarabine where we included '*guanine arabinoside*' in the search term as well. The total number of publications is shown in red bars on the left y-axis, the FDA approval year is shown in the blue line on the right y-axis. The cut-off for inclusion was 17th of July 2024.

B) We extracted all papers related to nelarabine or its precursors using the R package easyPubMed 2.13 as well, with search term '*Nelarabine OR 2-amino-6-methoxypurine arabinoside OR 2-amino-9-beta-D-arabinofuranosyl-6-methoxy-9H-purine OR 506U78 OR Arranon OR compound 506U78 OR GW506U78 OR 9 beta-arabinosylguanine OR 9 beta-D-arabinofuranosylguanine OR 9-arabinofuranosylguanine OR 9-arabinofuranosylguanine, (alpha)-isomer OR guanine arabinoside*'. The cut-off for inclusion was 17th of July 2024. All papers were then categorized based on the type of research, determined by reading of the abstract by the authors. The categories were: i) no original research (i.e., review papers or papers), ii) preclinical studies, iii) clinical studies (including retrospective studies), iv) case studies. Papers that were not accessible through the Karolinska Library or with no English abstract available were labelled as "not available". The pie chart insert shows a summary of the categories with the number of papers in each category in brackets. The color coding in the bar chart is the same as the pie chart.

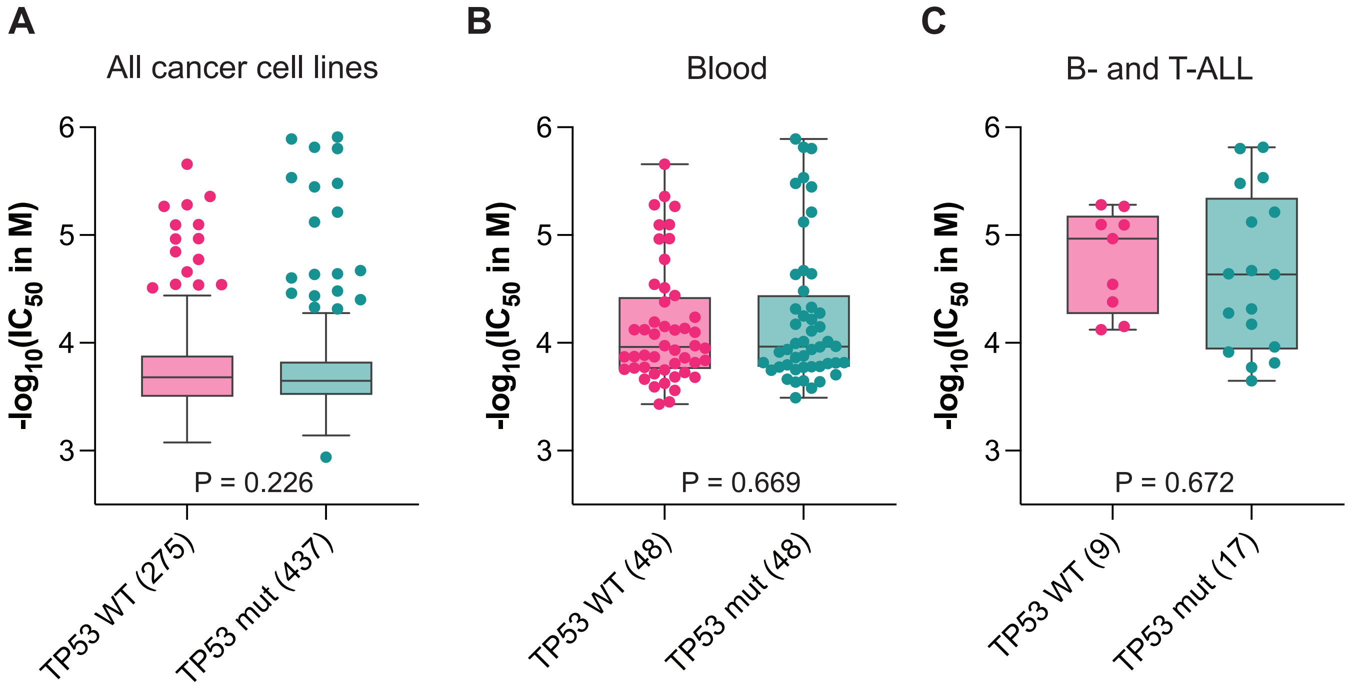

Supplemental Fig 2: Nelarabine sensitivity in p53 wildtype and mutated cell lines.
Nelarabine sensitivity of all cell lines in the Genomics of Drug Sensitivity in Cancer (GDSC) database [113] with known p53 mutation status. Data is divided based on mutation status in all cancer cell lines (A), blood cancer cell lines (B), or ALL cell lines (C). All cell lines with a probability of homozygous mutation over 50% were counted as mutated. Number in brackets shows the number of cell lines per group. A) Data is shown as $-\log_{10}(IC_{50}$ of nelarabine in M), box shows the median and 25-75 quartile and whiskers are plotted according to the Tukey method, outliers are shown as dots. B-C) All datapoints are shown, box shows the median and 25-75 quartile and the whiskers represent minimum to maximum value. A-C) P-value is calculated using the two-sided Mann-Whitney U-test on the $-\log_{10}(IC_{50})$ values.
